# Exercise Stress Echocardiography in Kawasaki Disease Patients with Coronary Aneurysms

**DOI:** 10.1007/s00246-022-03037-1

**Published:** 2022-11-16

**Authors:** Bruke A. Tedla, Jane C. Burns, Adrianna H. Tremoulet, Chisato Shimizu, John B. Gordon, Howaida El-Said, Fraser Golding, Christopher K. Davis, Kirsten B. Dummer

**Affiliations:** 1 Division of Pediatric Cardiology, Department of Pediatrics, Rady Children’s Hospital and University of California San Diego, 3020 Children’s Way, San Diego, CA USA; 2 Department of Pediatrics, Kawasaki Disease Research Center, Rady Children’s Hospital and University of California San Diego, 9500 Gilman Dr., La Jolla, CA USA; 3 San Diego Cardiac Center, 3131 Berger Ave, San Diego, CA 92123 USA

**Keywords:** Kawasaki, Stress echocardiography, Coronary aneurysms

## Abstract

The most significant sequelae of Kawasaki disease (KD) are coronary artery aneurysms, which can lead to risk of future myocardial ischemia. Exercise stress echocardiography allows for non-invasive assessment of myocardial dysfunction. We reviewed our single center experience with exercise stress echocardiography in patients with previous history of KD with coronary aneurysms. We reviewed the records of 53 KD patients who underwent exercise stress echocardiography from 2000 to 2020. Abnormal stress echocardiograms were defined as those showing no increase in biventricular systolic function post-exercise or regional wall motion abnormalities. Computed tomography angiography and cardiac magnetic resonance imaging were reviewed for patients with abnormal stress echocardiograms. Clinical data were reviewed and correlated with stress echocardiogram results. Of the 53 patients, three (5.7%) had an abnormal exercise stress echocardiogram. All three patients were classified as AHA Risk Level 4 or 5 by coronary Z-score (internal dimension normalized for body surface area) and were confirmed to have coronary aneurysms, stenosis, or myocardial tissue perfusion defects on advanced cardiac imaging that could account for the results seen on stress echocardiogram. Exercise stress echocardiography detected signs of myocardial ischemia in a subset of high-risk patients with Kawasaki disease and coronary aneurysms and may be considered as a useful screening tool for this complex patient cohort.

## Background

Kawasaki disease (KD) is the leading cause of acquired heart disease in developed countries and can lead to coronary artery aneurysms in ~ 25% of untreated cases [1–3]. Despite increasing awareness of KD and advances in medical therapies, approximately 5% of patients develop coronary aneurysms and 1% develop giant aneurysms [1, 4, 5]. Published guidelines (American Heart Association (AHA) and Japanese Circulation Society) provide guidance on the long-term management of KD patients, but they do not specify which tests should be used to assess for myocardial ischemia [2, 6, 7]. In the AHA guidelines, the assessment for inducible myocardial ischemia may include chemical or exercise stress coupled with echocardiography, electrocardiography, magnetic resonance (MR) perfusion imaging, or nuclear medicine perfusion imaging. The AHA guidelines recommend stress echocardiography for the following risk level patients at specified intervals: Risk Level 2, every 3–5 years; Risk Level 3, every 2–3 years; Risk level 4 (small aneurysm), every 1–3 years; Risk Levels 4 and 5 (large to giant aneurysms), every 6–12 months [2].

Exercise stress echocardiography in adults has a high sensitivity and specificity compared to other techniques such as single-photon emission computed tomography (SPECT) in identifying hemodynamically significant restrictions in coronary blood flow [8]. There have been comparatively fewer data in children with coronary artery disease, and the literature on KD using stress echocardiography is limited [9–14]. We reviewed our single center experience with exercise stress echocardiography in patients with a previous history of KD and coronary aneurysms to determine the yield of positive findings in these potentially at-risk groups.

## Methods

All subjects were enrolled in a prospective study of cardiac outcomes at a single tertiary care pediatric center from 2000 to 2020 and data were entered into a REDCap database. We reviewed data on 53 KD patients who underwent exercise stress echocardiography. The indications for exercise stress echocardiography included a history of KD with coronary aneurysms or dilation (Risk Levels 2–5), or other clinical concerns for potential ischemia. Patients underwent exercise stress testing via Bruce treadmill protocol or bicycle ergometry with imaging at rest and immediately post-exercise. Abnormal stress echocardiograms were defined as those showing no increase in biventricular systolic function post-exercise or regional wall motion abnormalities. Patients with abnormal stress echocardiograms underwent computed tomography angiography (CTA) or cardiac magnetic resonance imaging (CMRI). Echocardiographic measurements of the left anterior descending (LAD) and the right coronary arteries (RCA) were reviewed. The coronary arteries were measured following standard pediatric echocardiography protocols for the proximal, mid and distal portions when visible, and Dallaire Z-scores (internal dimension of the coronary artery normalized for body surface area and expressed as standard deviation units from the mean) were calculated [15]. The study was approved by the Institutional Review Board at the University of California San Diego (UCSD #140220) and parents and subjects signed consent and assent documents as appropriate.

Continuous variables were compared using Wilcoxon Rank Sum test and proportions were compared using Fischer’s exact test.

## Results

The clinical and demographic data for the study cohort are summarized in Table 1. All 53 patients were able to safely complete the study, and images were considered of diagnostic quality. Of the 53 patients, 48 were classified as AHA Risk Level 2–5. The remaining five patients had stress echocardiograms for other clinical indications: two patients had an anomalous right coronary, one patient had an anomalous left coronary artery, one patient had a short PR interval on electrocardiogram, and the final patient did not have a history of KD-associated coronary disease, but the test was performed at the specific request of the patient’s family.

**Table 1:** Clinical and demographic characteristics of the of study population

	Normal stress echo *n* = 50	Abnormal stress echo *n* = 3
Age at diagnosis (years)	1.3 (0.6–2.8)	2.1 (2.8–4.8)
Male, *n* (%)	38 (76%)	3 (100%)
Race/ethnicity		
Asian	15 (30.0)	
Black or African American	1 (2.0)	
White	12 (24.0)	
Multi-race	4 (8.0)	1 (33.0)
Hispanic	18 (36.0)	2 (66.0)
Illness day at diagnosis* (days)	6 (5–8)	10 (9.5–10)
Illness day at first IVIG treatment (days)	6 (5–10)	10 (9.5–10.5)
Age at first stress echocardiogram (years)	13.1 (10.4–15.3	10.3 (9.7–11.8)
Interval between age of diagnosis and first stress echocardiogram (years)	6.2 (3.5–7.5)	7.9 (5.7–8.3)
Number of stress echocardiograms per patient	1 (1–2)	2 (2–3)

Data are median (Interquartile range 25%–75%) or *n* (%)

*Illness Day 1 = first calendar day of fever

**Fig. 1:**
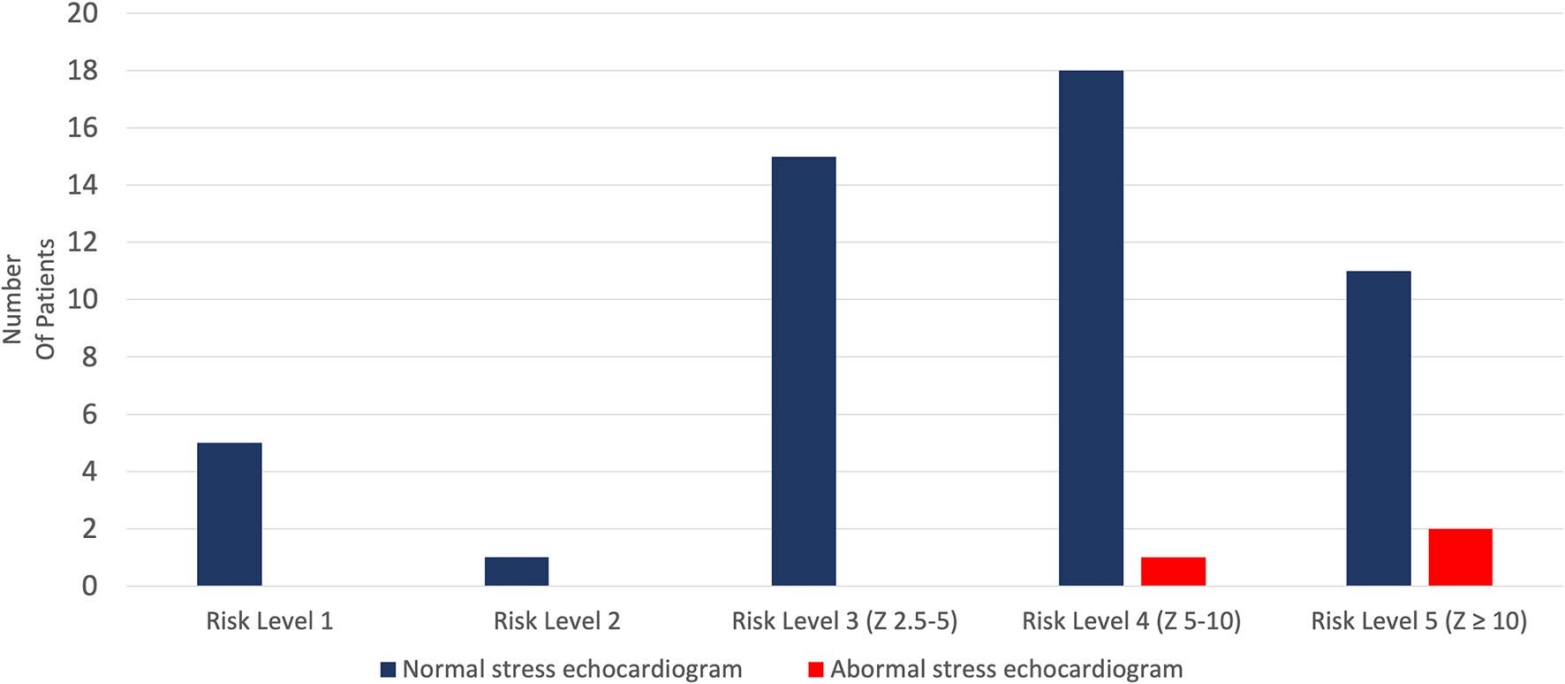
Patient risk level categories and exercise stress echocardiography. Distribution of patients with stress echocardiograms stratified by AHA Risk Level 1–5

Of the 32 patients classified as AHA Risk Level 4 and 5, three patients (9.4%) had abnormal exercise stress echocardiograms, which led to advanced imaging with CTA or CMRI (Fig. 1). All three patients had abnormal CTAs (coronary artery calcifications, aneurysm, or discrete coronary stenoses or occlusion) and one of the patients studied had an abnormal CMRI with myocardial perfusion defects (Table 2). Patient 1 had three abnormal stress echocardiograms (14 years, 15 years, and 17 years post-diagnosis) demonstrating a hypokinetic and mildly echo-bright inferior left ventricular free wall at rest. Post-exercise the same region of the left ventricle (LV) remained akinetic, but there was normal global increase in LV function despite the regional wall abnormalities. Patient 2 had one abnormal stress echocardiogram 8 years post-diagnosis demonstrating decreased thickening and systolic motion of the interventricular septum compared with the left ventricular posterior/lateral walls after exercise, followed by a normal stress echocardiogram 9 years after diagnosis. Patient 3 had one normal stress echocardiogram 4 years after diagnosis, followed by an abnormal stress echocardiogram 6 years after diagnosis demonstrating regional wall motion abnormality of the interventricular septum toward the apex and decreased thickening compared to the surrounding myocardium.

**Table 2:** Advanced imaging of patients with abnormal stress echocardiograms

Patient	Age of diagnosis (years)	Zmax LAD	Zmax RCA	AHA risk level	Exercise stress echocardiogram	Cardiac MRI	Cardiac CT	Clinical status
Patient 1	2 yo	(+ 23.2)	(+ 10.9)	Risk Level 5	At rest, there is a portion of the mid-basal inferior septum that is echobright and akinetic. Post-exercise, the same area of the left ventricle remains akinetic. There is normal increase in global LV systolic function post-exercise despite the regional wall abnormalities	Abnormal CMRI-Segmental akinesis of the mid, inferior LV free wall with full thickness hyper enhancement and abnormal first pass perfusion consistent with prior myocardial infarction	Abnormal CTA- Dense calcification of a small aneurysm in the mid RCA. Proximal RCA is small and occluded. Right IMA graft is patent	On aspirin, lisinopril, atorvastatin, nadolol, and amlodipine. ICD placed for VT arrest. Follows with adult KD team
Patient 2	4 yo	(+ 7.6)	(+ 7.1)	Risk Level 4	Global left ventricular systolic function increases normally with exercise. There is less thickening and systolic motion of the interventricular septum compared with the LV posterior and lateral walls post-exercise	Normal CMRI	Abnormal CTA- Aneurysmal segments demonstrated in all 3 major coronary arteries The largest aneurysm measures 6.4 mm × 8.0 mm at the ostium of the left circumflex artery. No stenosis	On aspirin, plavix, and atorvastatin. Follows annually with KD team
Patient 3	5 yo	(+ 19.2)	(+ 14.5)	Risk Level 5	Post-exercise, there is a regional wall motion abnormality of the interventricular septum toward the apex characterized by abnormal motion and decreased thickening compared with surrounding myocardium	None performed	Abnormal CTA- Overall increase in size of the fusiform aneurysms of the right coronary artery and left anterior descending coronary arteries	On atorvastatin, aspirin, and apixaban. Follows annually with KD team

### Patient Profiles

**Fig. 2:**
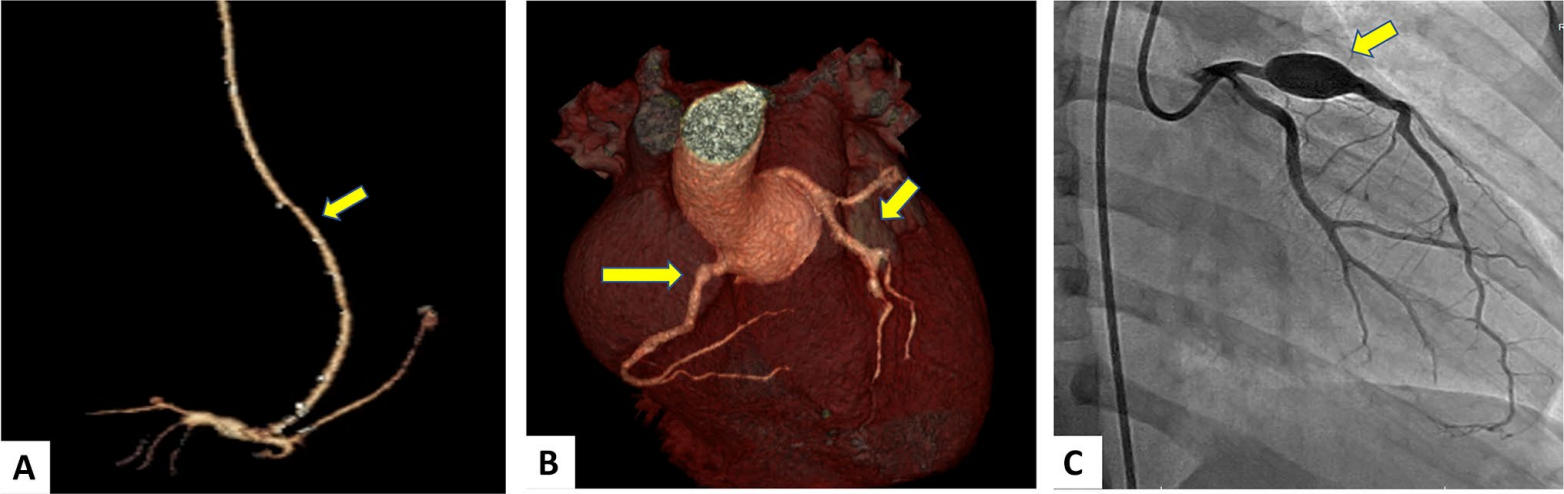
Coronary artery imaging in three patients with abnormal stress echocardiograms. **A** Patient 1 with CTA performed in 14 years after KD diagnosis demonstrating a patent RIMA to RCA bypass (yellow arrow). **B** Patient 2 with CTA performed 9 years after diagnosis demonstrating multiple coronary aneurysms (yellow arrows). **C** Patient 3 with cardiac catheterization performed 6 years after diagnosis demonstrating a giant coronary aneurysm in LAD (yellow arrow)

**Fig. 3:**
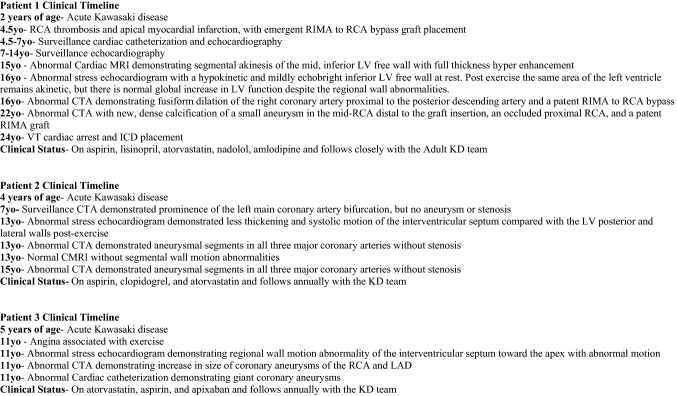
Clinical timelines of patients with abnormal stress echocardiograms

Patient 1 had KD at 2 years of age with an RCA Z max of + 23.2 and LAD Z max + 10.9 (AHA Risk level 5). At age 4.5 years, he developed thrombotic occlusion of the right coronary artery with an apical myocardial infarction. He emergently underwent coronary artery bypass grafting of the right internal mammary artery to the right coronary artery. He had been followed by a generalist and was on no medical therapy. Subsequently at 8 years of age, he was followed by the KD team and maintained on aspirin, lisinopril, and atorvastatin. He had surveillance cardiac catheterizations annually for the first 2 years and was followed with transthoracic echocardiography every 6 months, demonstrating stable, mildly diminished LV systolic function with LV EF 51%. Exercise stress echocardiogram 14 years after diagnosis demonstrated a hypokinetic and mildly echo-bright inferior LV free wall at rest. Post-exercise the same area of the left ventricle remained akinetic, but there was normal global increase in LV function despite the regional wall abnormalities. He remained active with participation in recreational soccer, basketball, and swimming without clinical signs of angina. He was later started on nadolol for ventricular ectopy and ventricular couplets, and amlodipine to optimize blood pressure. Twenty years after his initial myocardial infarction, he developed ventricular tachycardia with subsequent cardiac arrest. He was successfully resuscitated, and an implantable cardioverter defibrillator (ICD) was placed. He remains on aspirin, lisinopril, atorvastatin, nadolol, and amlodipine and has continued long-term follow up with the KD team (Figs. 2 and 3).

Patient 2 had KD at 4 years of age with coronary aneurysms with RCA Z max + 7.6 and LAD Z max + 7.1, classified as AHA Risk Level 4. He had a surveillance CTA 3 years after diagnosis that demonstrated prominence of the left main coronary artery bifurcation, but no aneurysm or stenosis. He was an active soccer player with no complaints of angina. Stress echocardiography performed 9 years after diagnosis demonstrated baseline normal LV function with less thickening and systolic motion of the interventricular septum compared with the LV posterior and lateral walls post-exercise. The abnormal stress echocardiogram findings correlated with the myocardial distribution of his coronary artery lesions. CTA demonstrated aneurysmal segments in all three major coronary arteries with the largest aneurysm measuring 6.4 mm × 8.0 mm (Z-score + 7.2) at the ostium of the left circumflex artery without stenosis. He remained on antiplatelet therapy with aspirin and clopidogrel and was started on atorvastatin to promote endothelial cell homeostasis [16, 17] (Figs. 2 and 3).

Patient 3 had KD at 5 years of age with giant coronary aneurysms with LAD Z max + 19.2 and RCA Z max + 14.5 (AHA Risk Level 5). Six years after diagnosis, he had possible angina associated with vigorous exercise while playing baseball, and underwent exercise stress echocardiography, CTA, and cardiac catheterization. Exercise stress echocardiography showed normal biventricular systolic function at rest but demonstrated regional wall motion abnormality of the interventricular septum toward the apex with abnormal motion and decreased thickening compared with the surrounding myocardium. The abnormal stress echocardiogram findings correlated with the distribution of his coronary artery lesions. CTA demonstrated an overall increase in size of the fusiform aneurysm of the RCA from 5.5 mm × 5.4 mm (Z-score + 8.2) to 6.4 mm × 6.4 mm (Z-score + 8.5), and an increase in the proximal LAD aneurysm from 7.1 × 7.2 mm (Z + score 14.0) to 9.7 × 9.3 mm (Z-score + 17.2). Cardiac catheterization was performed to further evaluate the coronary arteries and obtain hemodynamic measurements. The study confirmed large aneurysms in the proximal RCA and LAD without evidence of stenosis or obstruction. Cardiac index was 5.5L/min/m^2^, mean PA pressure 18 mm Hg, right ventricular pressure 30 mm Hg, and elevated left ventricular end diastolic pressure of 14 mm Hg against systolic systemic pressure 85 mm Hg. He was treated with aspirin, apixaban, and atorvastatin (Figs. 2 and 3).

During the review period, there were 50 patients with normal exercise stress echocardiograms. There were 18 patients who had a CTA performed, with three patients with abnormal CTAs notable for coronary artery calcifications, aneurysm, discrete coronary stenoses or occlusion. There were five patients who had a cMRI performed, which were negative for myocardial perfusion defects or wall motion abnormalities.

## Discussion

KD causes significant morbidity worldwide and can be associated with major adverse cardiac events including myocardial infarction and sudden death [1–5]. The most notable long-term complication is coronary artery aneurysm with or without remodeling that leads to increased risk of stenosis and thrombosis. In our series, three (9.4%) patients classified as AHA Risk level 4–5 had abnormal wall motion during stress echocardiography indicating ischemic changes with exercise. Conversely, none of 16 patients classified as AHA Risk level 2 or 3 had evidence of ischemia on stress echocardiography (Fig. 1).

Testing recommendations are based on risk stratification using echocardiographic Z-scores of coronary arteries [2, 15]. Once categorized, patients may be monitored for ischemia with functional tests including stress echocardiography and nuclear medicine perfusion imaging. Testing options however have different risks and requirements. Nuclear medicine perfusion imaging exposes the patient to repeated radiation (range 9–35 mSV per study), which is problematic in children given enhanced vulnerability to ionizing agents and increased carcinogenic risk profile compared to adults [18]. Though informative, the imaging modality has a lengthy acquisition time and often requires anesthesia and sedation in young children. CMRI can be helpful in identifying myocardial dysfunction and wall motion abnormalities, but the acquisition is typically obtained when the patient is at rest or under sedation and does not evaluate cardiac performance under stress/activity. The modality also requires peripheral IV placement and contrast media such as gadolinium, which can be contraindicated in certain patients. Exercise stress echocardiography, however, evaluates cardiac performance both at rest and activity, does not require sedation or IV placement, and does not expose the patient to radiation or contrast agents. Exercise stress echocardiography is limited by the age of the patient and ability to participate in the study, which is usually performed in children 6 years and older [13].

In our review, all three patients with abnormal stress echocardiography were classified as AHA Risk Level 4 or 5 by coronary Z- score and were confirmed to have abnormalities on CTA or CMRI that could account for the results on stress echocardiography. These results suggest that coronary blood flow hemodynamics during exercise are likely disturbed in severely dilated coronary arteries, which have undergone extensive remodeling in KD. Exercise stress echocardiography is a useful, noninvasive imaging modality to detect signs of myocardial ischemia in a subset of high-risk patients with KD and coronary aneurysms. For AHA Risk Level 1–3 patients, exercise stress echocardiography may be lower yield in screening for myocardial ischemia in KD patients during a follow up period of 10 years. If larger scale studies confirm these results in the future, the current AHA recommendation for routine use of exercise stress echocardiography in AHA Risk Levels 2 and 3 patients should be revisited.

We recognize several limitations to our study. The small number of patients studied precluded the ability to make robust conclusions regarding which patients should undergo stress echocardiography and the optimal timing and frequency of such testing. Future studies across multiple institutions will be necessary to develop best practice for stress echocardiography.
